# Sequence Context‐Agnostic TadA‐Derived Cytosine Base Editors for Genome‐Wide Editing in Zebrafish

**DOI:** 10.1002/advs.202411478

**Published:** 2025-02-17

**Authors:** Shaohui Zheng, Yang Liu, Xinxin Xia, Jiawang Xiao, Hui Ma, Xuanyao Yuan, Yan Zhang, Zixi Chen, Guangcong Peng, Wenyuan Li, Ji‐Feng Fei, Yanmei Liu

**Affiliations:** ^1^ Key Laboratory of Brain Cognition and Education Sciences Ministry of Education South China Normal University Guangzhou 510631 China; ^2^ Institute for Brain Research and Rehabilitation and Guangdong Key Laboratory of Mental Health and Cognitive Science South China Normal University Guangzhou 510631 China; ^3^ Department of Pathology Guangdong Provincial People's Hospital (Guangdong Academy of Medical Sciences) Southern Medical University Guangzhou Guangdong 510080 China; ^4^ China Zebrafish Resource Center (CZRC) Institute of Hydrobiology Chinese Academy of Sciences Wuhan 430072 China; ^5^ The Innovation Centre of Ministry of Education for Development and Diseases School of Medicine South China University of Technology Guangzhou 510006 China; ^6^ School of Basic Medical Sciences Southern Medical University Guangzhou 510515 China

**Keywords:** base editor, disease model, genome editing, zebrafish, zTadA‐CBEs

## Abstract

Single‐nucleotide variants (SNVs) represent a significant form of genetic variation linked to various diseases. CRISPR‐mediated base editing has emerged as a powerful method for modeling diseases caused by SNVs, particularly in zebrafish, which serve as an excellent platform for investigating disease mechanisms and conducting drug screenings. However, existing cytosine base editors (CBEs) for zebrafish often have broad editing windows and strong sequence preferences, limiting their effectiveness. In this study, zebrafish (z) TadA‐derived cytosine base editors, termed zTadA‐CBEs, are developed by introducing key mutations into the TadA8e enzyme. These novel editors demonstrate improved efficiency and precision in cytosine base editing. Specifically, zTadA‐BE4max and zTadA‐BEmv offer complementary editing windows, while zTadA‐SpRY‐BE4max allows for PAM‐flexible editing. Using zTadA‐CBEs, a precise disease model for Axenfeld‐Rieger syndrome is established, and created two new models for Hermansky‐Pudlak syndrome. Additionally, a novel albinism model carrying two pathogenic SNVs in the F0 generation is developed. By employing specifically designed sgRNA, the *fms^ts±^* missense mutation is corrected back to the wild‐type nucleotide (C > T), successfully restoring macrophage levels to normal. These findings underscore the potential of zTadA‐CBEs to enhance genome editing techniques and their applications in developing therapies for SNV‐related disorders.

## Graphical abstract

zTadA‐CBEs, developed by introducing key mutations into TadA8e, enable efficient and precise sequence context‐agnostic cytosine base editing. Specifically, zTadA‐BE4max and zTadA‐BEmv provide complementary editing windows, while zTadA‐SpRY‐BE4max offers PAM‐flexible editing. These tools establish various disease models in zebrafish, including a novel albinism model containing two pathogenic SNVs in the F0 generation, highlighting their potential for modeling polymorphic diseases.

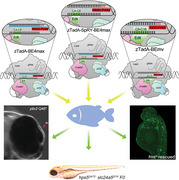

## 1 Introduction

Single nucleotide variants (SNVs) constitute over 96% of the observed genetic variation among humans.^[^ ^1^ ^]^ It is crucial to develop precise animal models harboring specific SNVs to investigate the potential pathogenic effects and explore possible treatments. Zebrafish, in particular, have emerged as an ideal model for genetic disease research as well as toxicological studies^[^ ^2^ ^]^ due to their small size, prolific breeding, transparent embryos, and significant genetic similarity to humans.

The advent of the CRISPR‐Cas9 system has transformed the speed, simplicity, and effectiveness of genome editing.^[^ ^3^ ^]^ Targeting of a genetic site with Cas9 necessitates pairing a single guide RNA (sgRNA) with the target DNA, along with recognition of the protospacer adjacent motif (PAM) located immediately downstream of the target site (e.g., NGG for Streptococcus pyogenes Cas9). Following the induction of a double‐stranded DNA break (DSB) by Cas9, the cell's DNA repair mechanisms commonly result in random insertions or deletions (indels) at the break sites, frequently leading to gene disruption. Achieving the precise introduction of specific point mutations at the target site through homology‐directed repair remains notably inefficient.

CRISPR/Cas9‐based base editors combine Cas9 protein with deaminases or other enzymes to chemically convert one nucleotide to another within the Cas9‐defined “editing window”, allowing for precise SNV mimicry or rescue.^[^ ^4^, ^5^, ^6^, ^7^ ^]^ Cytosine base editors (CBE), which convert a C•G base pair into a T•A pair, and adenine base editors (ABE), which convert an A•T base pair into a G•C pair, are widely used in both plant and animal systems.^[^ ^8^, ^9^, ^10^, ^11^ ^]^ In addition to CBEs and ABE, researchers have developed additional tools such as GBE/CGBE^[^ ^12^, ^13^ ^]^ for C to G conversion, gGBE^[^ ^14^ ^]^ for G to C/T conversion, AXBE and ACBE^[^ ^15^ ^]^ for A to the other three base conversions, AYBE^[^ ^16^ ^]^ for A to C/T conversion, and eTDG^[^ ^17^ ^]^ for T to G/C conversion as well as adenine‐ and cytosine‐dual‐base editors^[^ ^18^, ^19^, ^20^ ^]^ for versatile editing capabilities.

C•G to T•A transitions, primarily caused by the spontaneous deamination of cytosine, account for approximately half of all known human pathogenic SNVs^7^, and CBEs should, in theory, be able to generate model organisms bearing these changes. However, most current CBEs used in zebrafish are developed based on the rat cytidine deaminase rAPOBEC1, which exhibits characteristics that lead to broad editing windows and strong sequence context preferences, enabling the editing of C in TC/AC rather than GC/CC motifs,^[^ ^4^, ^6^, ^7^, ^21^, ^22^, ^23^ ^]^ thus limiting targeting sites. Recently, taking advantage of both adenosine and cytidine deamination activity of the engineered TadA^*^ enzyme in ABEs, several groups have independently developed TadA‐derived CBEs.^[^ ^24^, ^25^, ^26^ ^]^ Notably, Li Dali group has demonstrated that the introduction of N46L in TadA8e eliminates its adenosine deamination activity; the resulting Td‐CGBEs based on this mutation exhibited significantly higher editing efficiency at CC and GC motifs than APOBEC1‐derived CGBE.^[^ ^24^ ^]^ This suggests the potential for creating TadA‐derived CBEs that overcome sequence context bias, enabling genome‐wide editing in zebrafish.

In this study, we successfully developed the three TadA‐derived CBEs, termed zebrafish TadA‐CBEs (zTadA‐CBEs), for genome‐wide cytosine editing in zebrafish independent of sequence context. First, we engineered zTadA‐BE4max by introducing several key mutations into TadA8e (N46L/V82S/Q154R/E27H/I49K) optimized for zebrafish codons and replacing the ancAPOBEC1 of zAncBE4max with the TadA8e variant. The zTadA‐BE4max demonstrates more efficient C to T conversions in CC and GC motifs within a 5‐nucleotide window (positions 4–8 distal to the PAM) compared to the state‐of‐the‐art zebrafish CBE, zAncBE4max. The second tool, zTadA‐SpRY‐BE4max, facilitates efficient PAM‐flexible cytosine editing across various sequence contexts within the same narrow editing window, with a PAM preference of NRN > NYN (where R  =  A or G, and Y  =  C or T). The third tool, zTadA‐BEmv, exhibits notable efficiency in the editing window (positions 9–16 distal to the PAM), complementing zTadA‐BE4max and enabling editing of cytosines near the PAM region. By utilizing these three complementary zTadA‐CBEs, characterized by relatively low indel ratios and off‐target effects, we established various disease models in zebrafish, including models for Axenfeld‐Rieger syndrome and Hermansky‐Pudlak syndrome. Additionally, we developed a novel albinism model carrying two pathogenic SNVs in the F0 generation. Using specifically designed sgRNA, we corrected the *fms^ts±^* missense mutation back to its wild‐type nucleotide (C>T), successfully restoring macrophage levels to normal. The introduction of these zTadA‐CBEs represents a significant advancement in zebrafish genome editing, paving the way for the development of additional TadA‐derived CBEs and facilitating the generation of comprehensive models for human SNV‐related diseases on a genome‐wide scale.

## 2 Results

### 2.1 The zTadA‐BE4max Demonstrates Efficient Cytosine Base Editing Across Various Sequence Contexts

To develop a sequence context‐agnostic TadA‐derived CBE with minimal adenosine deamination activity, we explored different combinations of mutations incorporating N46L in TadA8e, based on the previous studies.^[^ ^24^, ^26^, ^27^, ^28^, ^29^ ^]^ These various TadA8e variants were integrated at the N‐terminal of spCas9n (D10A) nickase, which was linked to two copies of uracil glycosylase inhibitors (UGIs) at the C‐terminal and a bipartite nuclear localization signal (bpNLS). All the sequences were optimized according to zebrafish codon preference (Figure S1A, Supporting Information). The resulting four constructs zTadA‐BE4max‐a (N46L), zTadA‐BE4max‐b (N46L/V82S/Q154R), zTadA‐BE4max‐c (N46L/E27H/I49K), and zTadA‐BE4max (N46L/V82S/Q154R/E27H/I49K) were assessed for cytosine editing efficiency at two sgRNA targeting sites, *rpl9*‐NGG and *rpl17*‐NGG. Following the injection of the individual mRNAs transcribed in vitro from these constructs, along with relevant 2′‐O‐methyl‐3′‐phosphorothioate (MS)‐modified gRNAs into one‐cell stage zebrafish embryos, we extracted genomic DNA at 48 h post‐fertilization (hpf) for PCR amplification and Sanger sequencing to analyze the base editing outcomes. The results revealed that only zTadA‐BE4max achieved over 40% cytosine editing efficiencies at both sites, while the other three tools exhibited editing efficiencies of less than 10% (Figure S1B, Supporting Information). Notably, no A to G substitutions were detected at the targeting site *rpl9*‐NGG using any of the four tools (Figure S1B, Supporting Information).

**Figure 1:**
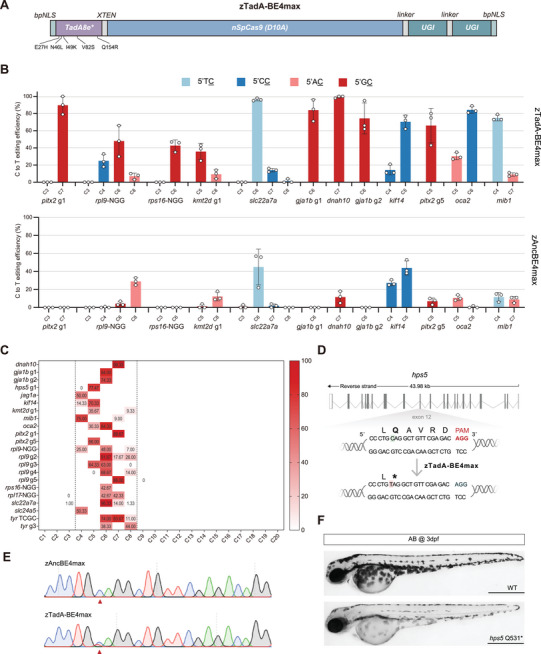
Efficient cytosine base editing in different sequence contexts mediated by zTadA‐BE4max. A) Schematic of the mRNA construct for zTadA‐BE4max. B) Comparison of the editing efficiency between zAncBE4max and zTadA‐BE4max targeting twelve different loci with NGG PAM. C) Summary of editing efficiency of zTadA‐BE4max at twenty‐two NGG PAM sites on fifteen genes. The editing window is outlined by two dashed lines, and the average editing efficiency value for each cytosine locus is shown. D) Schematic diagram of the *hps5* Q531^*^ target locus. The targeted sequence is shown with the PAM highlighted in red. The targeted cytosine nucleotide and expected changes are highlighted in green and red, respectively, while the targeted amino acid and expected changes in amino acid are indicated with bold text. E) Comparison of Sanger peak plots for zAncBE4max and zTadA‐BE4max at the *hps5* Q531^*^ locus. The red arrowhead indicates the expected nucleotide substitutions. F) Lateral view of 3dpf F1 homozygous embryos with the *hps5* (Q531^*^) mutation (bottom), exhibiting pigmentation defects compared to the wild‐type (top). Scale bar: 500 µm.

Subsequently, we concentrated on zTadA‐BE4max and evaluated its editing characteristics using additional sgRNAs targeting various loci (**Figure** **1**A). We selected twelve sgRNAs targeting loci *pitx2 *g1/g5, *rpl9*‐NGG, *rps16*‐NGG, *kmt2d* g1, *oca2*, *slc22a7a*, *kif14*, *dnah10*, *gja1b* g1/g2 and *mib1*, comparing the cytosine editing efficiency of zTadA‐BE4max with zAncBE4max. Within the zAncBE4max editing window, which encompasses two TC sites and four AC sites (located 4–8 positions distal to the PAM), both tools exhibited a range of efficiencies, demonstrating distinct advantages at specific sites. However, at the eight GC sites and five CC sites within the editing window, zTadA‐BE4max produced noticeable C‐to‐T base substitutions at all GC sites and at four CC sites, whereas zAncBE4max showed low cytosine editing efficiency at most of these sites (Figure 1B). The peak editing efficiency of zTadA‐BE4max at the GC site reached 99.33% ± 1.15%, significantly surpassing the mere 11.67% ± 6.50% peak editing efficiency of zAncBE4max at the same site. Across the five CC sites, the editing efficiency of zTadA‐BE4max varied from 14.00% ± 2.00% to 84.33% ± 4.16%, which represented an improvement of 12.00% to 83.67% compared to zAncBE4max at four of the CC sites. We examined the cytosine editing efficiency of zTadA‐BE4max across a total of twenty‐five targeting sites, noting that only three loci were edited below 10% efficiency, which we considered invalid. Further analysis of the twenty‐two effective loci for cytosine editing indicated that the primary editing window of zTadA‐BE4max corresponds with that of zAncBE4max (specifically positions 4–8 distal to the PAM) (Figure 1C). In summary, zTadA‐BE4max transcends the editing constraints of zAncBE4max for CC and GC sites, enabling the targeting of cytosines in any sequence context in zebrafish.

To effectively utilize zTadA‐BE4max, we designed an sgRNA to direct it to a pathogenic SNV site, aiming to create a zebrafish Hermansky‐Pudlak syndrome (HPS) disease model that could not be generated with previous effectors. HPS is a rare autosomal recessive genetic disorder characterized by oculocutaneous albinism (OCA) and a tendency to bleed due to defects in melanosome and platelet delta granule secretion.^[^ ^30^ ^]^ *HPS5*, encoding a subunit of BLOC2 (biogenesis of lysosome‐related organelles complex), has been reported as one of the pathogenic genes associated with HPS.^[^ ^31^, ^32^ ^]^ HPS5 exhibits a high degree of conservation with zebrafish hps5, with amino acid homologies of 74.0% (Figure S2, Supporting Information). To create a zebrafish model of HPS that mimics the human HPS5 SNV: c.1618C>T (p. Q540^*^, NM_181507.1),^[^ ^33^ ^]^ we designed an sgRNA targeting the homologous site in zebrafish to install the *hps5* c.1591C>T (p. Q531^*^) mutation using zTadA‐BE4max (Figure 1D). Consistent with our previous findings, this target cytosine was located in a GC sequence context, rendering zAncBE4max ineffective at this site (Figure 1E). Remarkably, the editing efficiency of zTadA‐BE4max at this site was ≈30% (Figure 1E). The pigmentation of homozygous juveniles carrying the hps5 ^Q531*^ mutation in the F1 generation displayed a significantly lighter hue, thereby confirming the pathogenicity of the SNV in the zebrafish model (Figure 1F). These results highlight the robust application of zTadA‐BE4max as a superior base editor capable of overcoming sequence context preferences for disease modeling in zebrafish.

The majority of previous zebrafish genetic disease models have focused on single‐gene pathogenicity studies, with more complex polygenic composite mutations rarely being explored due to the challenges associated with constructing polymorphic disease models. To verify whether zTadA‐BE4max can effectively create polymorphic disease models, we aimed to simultaneously target two loci associated with HPS: *slc24a5* Q74^*^ and *hps5* Q473^*^. The results demonstrated that hps5^Q473*^slc24a5^Q74*^ F0 zebrafish displayed a more pronounced albinism phenotype, possibly due to the cumulative effects of the double mutations in *hps5* and *slc24a5* (Figure S3 AA’‐DD’, Supporting Information). The hps5^Q473*^slc24a5^Q74*^ F0 fish achieved similar editing efficiencies at both the *hps5* Q473^*^ and *slc24a5* Q74^*^ loci in comparison to the single mutation F0 models (hps5^Q473*^ or slc24a5^Q74*^) (Figure S3E, Supporting Information). Our findings indicate that zTadA‐BE4max is capable of effectively targeting at least two loci simultaneously, which may be beneficial for constructing polymorphic disease models and advancing drug screening.

### 2.2 The zTadA‐SpRY‐BE4max Exhibits High Activity Across Various Sequence Contexts at Non‐Canonical PAM Sites

**Figure 2:**
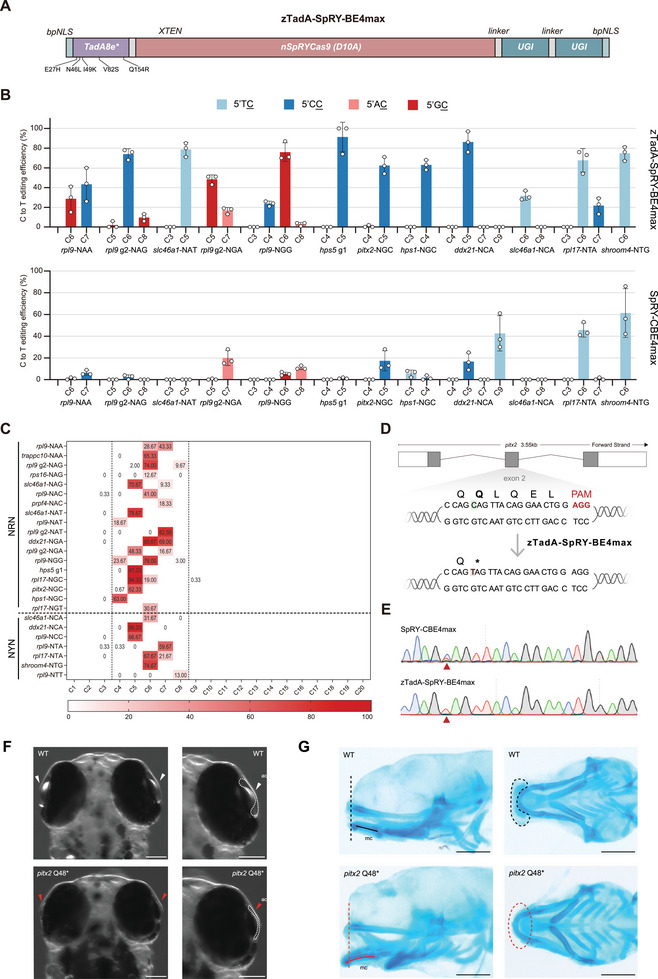
Efficient cytosine base editing in various sequence contexts targeting non‐canonical PAMs by zTadA‐SpRY‐BE4max. A) Schematic of the mRNA construct for zTadA‐SpRY‐BE4max. B) Comparison of the editing efficiency between SpRY‐CBE4max (bottom) and zTadA‐SpRY‐BE4max (top) targeting twelve different loci with different PAMs. C) Editing efficiency of zTadA‐SpRY‐BE4max at twenty‐five NNN PAM sites across eleven genes. The editing window is outlined by two dashed lines, and the average editing efficiency value for each cytosine locus is shown. D) Schematic diagram of the *pitx2* Q48^*^ target locus. The targeted sequence is shown with the PAM highlighted in red. The targeted cytosine nucleotide and expected changes are highlighted in green and red, respectively, while the targeted amino acid and expected changes in amino acid are indicated with bold text. E) Comparison of Sanger sequencing results for SpRY‐CBE4max and zTadA‐SpRY‐BE4max at the *pitx2* Q48^*^ target locus. The red arrowhead indicates the expected nucleotide substitutions. F) Dorsal view of 5 dpf F1 homozygous embryos with the *pitx2* Q48^*^ mutation, displaying absence of anterior chamber (ac). White arrows and dotted lines indicate the intact anterior chamber structure, while the abnormally developed ac structure within the mutant is highlighted by red arrows and traced dotted lines. Scale bar: 100 µm. G) Alcian blue staining of 5 dpf wild‐type and *pitx2* Q48^*^ homozygous mutant embryos in ventral and lateral views. The black dotted line and the red dotted line indicate the position of the ethmoid plate and Meckel's cartilage in the wild type and the mutant, respectively. Scale bar: 200 µm.

To expand the sequence context‐agnostic advantages of zTadA‐BE4max to more targeting sites, we aimed to develop enhanced zTadA‐CBE capable of overcoming PAM restrictions. The most flexible SpCas9 variant, SpRYCas9, along with its related base editors, SpRY‐CBE4max, has been reported to target nearly all PAM sequences across the genomes of cultured cells, plants, and zebrafish.^[^ ^34^, ^35^, ^36^, ^37^, ^38^ ^]^ Recently, we and Rosello et al. independently reported that SpRY‐CBE4max can recognize almost all NRN PAM sequences, thereby expanding its potential to target previously inaccessible bases in zebrafish for base editing.^[^ ^37^, ^38^ ^]^ However, SpRY‐CBE4max also exhibits limitation in terms of sequence context preference, particularly for editing GC. Building upon our previous success in mitigating sequence context preference with zTadA‐BE4max, we subsequently developed zTadA‐SpRY‐BE4max by replacing the SpCas9n moiety with SpRYCas9n (**Figure** **2**A). We employed twelve sgRNAs targeting non‐canonical PAMs to assess the base editing activity of zTadA‐SpRY‐BE4max and SpRY‐CBE4max across every cytosine, regardless of sequence context. Our observations indicated that zTadA‐SpRY‐BE4max effectively induced C‐to‐T base conversions at nearly all CC and GC sites, demonstrating significantly higher editing efficiency compared to SpRY‐CBE4max (Figure 2B). To further characterize its editing window and PAM preference, we evaluated the editing efficiency of zTadA‐SpRY‐BE4max at seventeen additional targeting sites with varying PAMs. Among the tested ten NRN PAM sites, zTadA‐SpRY‐BE4max consistently displayed robust CBE activity, with editing efficiencies reaching as high as 94.33% ± 6.02%, nearly approximating homozygous mutation levels (Figure S4, Supporting Information). In contrast, among the seven NYN PAM sites selected, zTadA‐SpRY‐BE4max achieved efficient editing at only three sites, with the highest efficiency recorded at 66.67% ± 17.04%. The remaining four sites had base editing efficiencies below 10% (Figure S4, Supporting Information). These results indicate a preference for NRN PAMs over NYN PAM by zTadA‐SpRY‐BE4max, which is consistent with prior findings regarding SpRYCas9 and SpRY‐CBE4max. Furthermore, zTadA‐SpRY‐BE4max exhibited the same narrow editing window as that of zTadA‐BE4max (positions 4–8 distal to the PAM) (Figure 2C), which is one nucleotide narrower than the editing window of SpRY‐CBE4max (positions 4–9 distal to the PAM), suggesting greater precision in editing relative to SpRY‐CBE4max.

Next, we applied zTadA‐SpRY‐BE4max to create a precise disease model of Axenfeld–Rieger syndrome (ARS) in zebrafish. ARS is a clinically diverse autosomal‐dominant disorder characterized by anomalies in the anterior segment of the eyes, craniofacial and dental irregularities, cardiovascular malformations, and additional periaqueductal skin.^[^ ^39^ ^]^ The p.Q50^*^ mutation in PITX2 has been implicated in causing ARS.^[^ ^40^ ^]^ However, achieving the precise C‐to‐T conversion at the corresponding site c.142C (p.Q48^*^) of *pitx2* is challenging with previous CBEs due to its GC context (Figure S5, Supporting Information; Figure 2D). We designed a sgRNA targeting c.142C (p.Q48^*^) of *pitx2* to implement the C‐to‐T mutation using zTadA‐SpRY‐BE4max (Figure 2D). Remarkably, the editing efficiency of zTadA‐SpRY‐BE4max at this site was ≈76.67% ± 24.09% (Figure 2E). Homozygous pitx2 ^Q48*^ mutant zebrafish generated via zTadA‐SpRY‐BE4max editing exhibited underdeveloped anterior chambers (Figure 2F) and craniofacial deformities at 5 dpf (Figure 2G), mirroring the phenotypic traits seen in human ARS cases. Together, these findings demonstrate the potency of zTadA‐SpRY‐BE4max as an innovative tool for precise, effective, and PAM‐flexible base editing, free from sequence context biases, thereby paving the way for enhanced human genetic disease modeling in zebrafish.

Besides establishing disease models, we next investigated whether zTadA‐SpRY‐BE4max can correct mutations, such as in the *fms* gene, which encodes the Fms receptor, essential for macrophage development. In *fms^ts^* zebrafish, the Y185H substitution caused by an A>G change impairs macrophage development, leading to a significant decrease in their numbers at elevated temperatures (33 °C).^[^ ^41^ ^]^ Using designed sgRNA targeting the *fms* missense mutation, we used zTadA‐SpRY‐BE4max to edit embryos from *fms^ts+/−^* inter‐crosses. After editing, the recovery rate of macrophages in the injected embryos was significantly higher than in the uninjected controls, with 7 out of 8 injected embryos displaying normal macrophage counts (Figure S6, Supporting Information). These results confirm the potential of zTadA‐SpRY‐BE4max to effectively correct mutations, advancing developments in genome editing and therapeutic strategies.

### 2.3 The zTadA‐BEmv Demonstrates a Complementary Cytosine Editing Window Compared to Existing CBEs

**Figure 3:**
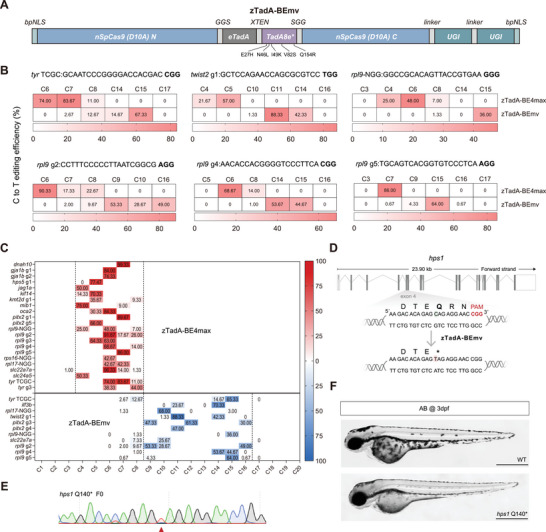
Complementary cytosine editing mediated by zTadA‐BEmv and zTadA‐BEmax. A) Schematic of the mRNA construct for zTadA‐BEmv. B) Comparison of the targeting window between zTadA‐BEmv (bottom) and zTadA‐BE4max (top) targeting six different loci with NGG PAM. C) Comparison of targeting window and editing efficiency between zTadA‐BEmax targeting twenty‐two NGG PAM sites across fifteen genes and zTadA‐BEmv targeting eleven NGG PAM sites across seven genes. The editing window of each tool is outlined by two dashed lines, and the average editing efficiency value for each cytosine locus is shown. D) Schematic diagram of the *hps1* Q140^*^ target locus. The targeted sequence is shown with the PAM highlighted in red. The targeted cytosine nucleotide and expected changes are highlighted in green and red, respectively, while the targeted amino acid and expected changes in amino acid are indicated with bold text. E) Sequencing results of F0 embryo after editing with zTadA‐BE4max. The red arrowhead indicates the expected nucleotide substitutions. F) Lateral view of 3 dpf F1 homozygous embryos with the *hps1* (Q140^*^) mutation (bottom), exhibiting pigmentation defects compared to the wild‐type (top). Scale bar: 500 µm.

While zTadA‐SpRY‐BE4max exhibits significant advantages over SpRY‐CBE4max, we observed that zTadA‐SpRY‐BE4max remains ineffective at certain NYN sites. Consequently, we developed a third tool, zTadA‐BEmv, which creates a complementary editing window under the NGG PAM. To shift the editing window of TadA8e closer to the PAM, we replaced the HNH domain of SpCas9 with a dimer of *E. coli* TadA (eTadA) and TadA8e, thereby constructing zTadA‐BEmv (**Figure** **3**A). To verify the changes in the editing window of zTadA‐BEmv, we selected six targeting sites containing multiple cytosines at positions 4 to 17 distal to the PAM for comparative injections of zTadA‐BE4max and zTadA‐BEmv. Additionally, the editing efficiency of zTadA‐BEmv was assessed at fifteen distinct loci, of which eleven showed editing efficiencies greater than 10%. The results showed that zTadA‐BEmv exhibited lower editing activity at C7 and C8, but demonstrated higher editing activity from C9 to C16, effectively complementing the editing window of zTadA‐BE4max (C4–C8) (Figure 3B,C). Importantly, zTadA‐BEmv maintained a significant editing efficiency within its primary editing window compared to zTadA‐BE4max (Figure 3B).

Next, we applied zTadA‐BEmv to establish a disease model for the pathogenic mutation NM_000195.5 (*HPS1*): c.421C>T (p.Q141^*^) in zebrafish. *HPS1* encodes a subunit of the BLOC‐3 protein, and mutations in the *HPS1* gene can disrupt the normal assembly of BLOC‐3, leading to the onset of HPS symptoms.^[^ ^42^, ^43^ ^]^ By aligning the amino acid and DNA sequence of *HPS1* and its zebrafish homolog *hps1* (Figure S5, Supporting Information), we designed an sgRNA targeting the corresponding site c.418C>T (p.Q140 *) of *hps1* to implement the C‐to‐T mutation using zTadA‐BEmv. The target cytosine resides in a GC motif at position C12 under the NGG PAM (Figure 3D). Remarkably, zTadA‐BEmv achieved efficient C‐to‐T editing at the C12 position, with an impressive efficiency of 85.00% ± 17.35% (Figure 3E). Although low bystander editing at C7 was detected, it can be easily excluded by genotyping individual embryos. We successfully identified homozygous mutant hps1^Q140*^ zebrafish exhibiting lighter pigmentation (Figure 3F), demonstrating the potential of the zTadA‐BEmv tool for achieving high‐efficiency editing and modeling human SNV‐related diseases.

### 2.4 The zTadA‐CBEs Exhibit High Product Purity and Low Off‐Target Effects

Given the potential practical challenges associated with their application, it is necessary to thoroughly characterize zTadA‐CBEs, particularly focusing on product purity and off‐target effects, before their widespread use in modeling human SNV‐related diseases. We have summarized the editing efficiencies of all cytosine sites we examined (including ineffective ones) within the editing range of zTadA‐CBEs, and this information will be useful for guiding the selection of zTadA‐CBEs in specific contexts (Figure S8, Supporting Information). To this end, we PCR‐amplified the zTadA‐CBEs edited sequences at the three sgRNA targeting sites relevant to zebrafish disease models: hps5 ^Q531*^, pitx2 ^Q48*^, and hps1^Q140*^. These sequences were subjected to next‐generation sequencing (NGS) analysis to evaluate the indel ratio and product purity. Additionally, we PCR‐amplified the sequences surrounding the three most probable off‐target sites for each sgRNA, as predicted by CRISPOR,^[^ ^44^ ^]^ for NGS analysis to investigate the potential off‐target effects of these 3 zTadA‐CBEs. We detected relatively low levels (10.96%, 4.67%, and 1.19%) of indels and undesired C‐to‐A or C‐to‐G conversions (0.56%, 0.54%, and 1.68%) at each of the sgRNA targeting sites edited using zTadA‐BE4max, zTadA‐SpRY‐BE4max and zTadA‐BEmv, respectively (**Table** **1**). The off‐target editing rates for zTadA‐BE4max, zTadA‐SpRY‐BE4max, and zTadA‐BEmv ranged from 0 to 0.02%, 0 to 0.09%, and 0.01 to 8.49%, respectively (Table 1). These findings indicate that the three zTadA‐CBEs developed in this study contribute to a relatively low indel ratio, high product purity, and minimal off‐target effects. Moreover, zTadA‐CBEs exhibit significantly high germline targeting efficiency and germline transmission rates (Table S1, Supporting Information). Collectively, these characteristics position zTadA‐CBEs as powerful tools for establishing genome‐wide zebrafish models relevant to human SNV‐related diseases.

**Table 1:** Assessment of On‐Target and Off‐target editing by zTadA‐CBEs using NGS. The target cytosine and PAM sequences are indicated with bold text.

gRNA	Tools	Mismatch Location	Sequence	MitOfftarget Score	Reads	Efficiency	Indel	Incorrect Editing
*hps5* Q531* sgRNA	zTadA‐BE4max	………………	CCCTG**C**AGGCTGTTCGAGAC**AGG**	–	1135098	C3T, 0.14%; C6T, 31.02%; C10T, 0.05%	10.96%	0.56%
		.*…*.*……*……	CACTGTAAGCTGTTAGAGAC**AGG**	0.25	705239	C3T, 0.00%; C10T, 0.00%	0.06%	0.00%
		*….*….*…*……	GCCTGGAGGCGGTTAGAGAC**AGG**	0.14	4450723	C2T, 0.00%; C3T, 0.01%; C10T, 0.02%;	0.00%	0.02%
		……*…*…*…*…	CCCTGGAGGGTGGTCGGGAC**CGG**	0.12	243647	C2T, 0.00%; C3T, 0.00%;	0.00%	0.00%
*pitx2* Q48* sgRNA	zTadA‐SpRY‐BE4max	…………………	CCAG**C**AGTTACAGGAACTGG**AGG**	–	497476	C2T, 0.13%; C5T, 70.18%	4.67%	0.54%
		*…*…………….	TCATCAGTTACAGGAACTGG**TAA**	5.72	716542	C2T, 0.00%; C5T, 0.00%	0.06%	0.00%
		…….*.*……….	CCAGCAGCTCCAGGAACTGG**AAG**	5.03	1022376	C2T, 0.00%; C5T, 0.09%; C8T, 0.00%; C10T, 0.00%	0.12%	0.01%
		.*…*…*…………	CAAGTAGCTACAGGAACTGG**AGC**	2.54	937729	C8T, 0.02%	0.17%	0.03%
*hps1* Q140* sgRNA	zTadA‐BEmv	…………………	AAGACACAGAG**C**AGAGGAAC**CGG**	–	827724	C5T, 4.31%; C7T, 25.67%; C12T, 72.14%	1.19%	1.68%
		…*……………*.	AACACACAGAGCAGAGGATC**TGG**	4.76	2877828	C3T, 0.04%; C5T, 0.62%; C7T, 3.57%; C12T, 8.49%	0.04%	0.05%
		………*…*…….	AAGACACAGCGCTGAGGAAC**GGG**	2.04	3146644	C5T, 0.02%; C7T, 0.05%; C10T, 0.10%; C12T, 0.31%	0.01%	0.00%
		…**……………*	AATCCACAGAGCAGAGGAAT**GGG**	1.38	5522304	C4T, 0.03%; C5T, 0.10%; C7T, 0.96%; C12T, 1.82%	0.00%	0.00%

## 3 Discussion

This study has developed three zTadA‐CBEs for zebrafish: zTadA‐BE4max, created by substituting the ancAPOBEC1 of zAncBE4max with the TadA8e variant (N46L/V82S/Q154R/E27H/I49K), zTadA‐SpRY‐BE4max, formed by replacing the SpCas9n of zTadA‐BE4max with SpRYCas9n; and zTadA‐BEmv, generated by substituting the HNH domain of SpCas9n in zTadA‐BE4max with a dimer of eTadA and TadA8e. All three zTadA‐CBEs demonstrate remarkably efficient C to T conversions in CC and GC motifs, a feat unattainable by conventional CBEs in zebrafish. Furthermore, zTadA‐SpRY‐BE4max exhibits adept PAM‐flexible cytosine editing, with a PAM preference of NRN > NYN, while zTadA‐BEmv achieves cytosine editing from C9 to C16 under NGG PAM, complementing the capabilities of zTadA‐BE4max and other CBEs. By utilizing these three zTadA‐CBEs, we established several disease models in zebrafish, including Axenfeld‐Rieger syndrome and Hermansky‐Pudlak syndrome, developed a novel albinism model with two pathogenic SNVs, and corrected the *fms^ts±^* missense mutation to rescue its phenotype. Additionally, we have demonstrated that using the three sgRNAs for these disease models, zTadA‐CBEs exhibit relatively low indel ratios and off‐target effects. This study marks the initial introduction of zTadA‐CBEs into zebrafish research, significantly enhancing the capacity to investigate disease pathogenesis stemming from SNVs and to screen potential drugs for human SNV‐related diseases.

The zTadA‐CBEs not only represent the first TadA8e‐derived CBE tools for modeling human pathogenic SNVs in zebrafish but also present significant potential for optimization. By incorporating TadA8e and its variants from ABEs into CBEs, we can apply the modification strategies of ABEs to enhance zTadA‐CBEs, leading to increased efficiency and more precise, controllable editing. For instance, the design of zTadA‐BEmv is inspired by a novel ABE tool, HNHxABEHD^[^ ^45^ ^]^ in which a monomeric or heterodimeric TadA replaces the HNH nuclease domain of SpCas9, shifting the editing window closer to the PAM. Moving forward, various optimizations of ABEs based on the TadA structure can be explored for potential application in zTadA‐CBEs.

The predominant CBE tool in zebrafish research, SpRY‐BE4max, known for its preference for TC editing, has an editing window of C4–C9^[^ ^37^ ^]^ or C3–C9.^[^ ^38^ ^]^ In contrast, our newly developed CBE, zevoCDA1‐198, which also facilitates effective editing of cytosines within GC and CC motifs, occasionally achieves significant cytidine editing at positions C‐1 or even C‐2, in addition to the primary editing window of C1–C5.^[^ ^46^ ^]^ The zTadA‐SpRY‐BE4max introduced in this study demonstrates a precise editing window of C4–C8. This represents a significant improvement in editing accuracy by narrowing the editing window by 1–2 nucleotides, making it the PAM‐flexible CBE with the narrowest editing window currently available.

Among spCas9n‐based CBE tools that recognize the NGG PAM, zTadA‐BE4max has an editing window of C4–C8, which is comparable to the C3–C7 window of zAncBE4max (we also observed notable editing efficiency at C8 with zAncBE4max).^[^ ^22^ ^]^ On the other hand, zTadA‐BE4max is more accurate than zevoCDA1‐BE4max, which has a broader editing window of C1–C9,^[^ ^46^ ^]^ Additionally, zTadA‐BEmv, with an editing window of C9–C16, is the first NGG PAM‐recognized CBE capable of achieving editing near the PAM region. This tool complements other spCas9n‐based CBEs, including zAncBE4max, zevoCDA1‐BE4max, and zTadA‐BE4max.

Importantly, zTadA‐CBEs overcome the CC/GC context limitation, which is a challenge that can only be addressed by zevoCDA1‐CBEs with a broader editing window. In contrast, zTadA‐SpRY‐BE4max, developed in this study, showcases a precise C4–C8 editing window, making it the SpRYCas9‐based CBE with the narrowest window currently available, thus facilitating enhanced precision in editing. Moreover, the complementary editing windows of zevoCDA1‐198 and zTadA‐SpRY‐BE4max enable easy identification of downstream high‐efficiency NRN PAM sequences in practical modeling scenarios. The synergistic use of these two tools has the potential to significantly improve the success rate of modeling various human SNV‐related diseases in zebrafish.

While the zTadA‐CBE toolkit represents a step forward in addressing some of the limitations of existing zebrafish CBE tools, the optimization brought by zTadA‐CBEs is still limited in the broader context of zebrafish CBE tools. There remains a demand for more robust and diverse CBEs to strengthen the field. ABEs have evolved into an advanced version, ABE9, with editing windows as narrow as 1–2 nucleotides.^[^ ^47^ ^]^ Recently, in collaboration with the Varshney group, we introduced a series of precise editing ABE‐ultramax tools for zebrafish: ABE‐Umax‐rest1 (with an editing window of A5‐A6), ABE‐Umax‐ex1‐rest1 (with an editing window of A4‐A6), and ABE‐Umax‐ex2‐rest1 (with an editing window of A12‐A15).^[^ ^48^ ^]^ These finely calibrated editing windows are flexible enough to cater to a range of modeling scenarios, thereby significantly enhancing the precision of SNV modeling in zebrafish research. In contrast to ABEs, zebrafish CBEs, which possess editing windows of at least 5 nucleotides, require further narrowing to elevate the editing accuracy and finesse within the field.

The breakthrough of SpRYCas9 in overcoming the NGG PAM limitation presents a double‐edged sword, offering both advantages and potential risks. While the expansion of the editing scope to include NNN PAMs greatly broadens the applicability of base editors, it also raises concerns regarding increased sgRNA‐dependent DNA off‐target effects. Although existing studies on SpRY‐BE4max^[^ ^37^, ^38^ ^]^ and the off‐target evaluation of zTadA‐SpRY‐BE4max have indicated minimal off‐target impacts when paired with specific sgRNAs, it is important to note that the possibility of significant off‐target effects with alternative sgRNAs cannot be definitively ruled out. Nevertheless, any potential erroneous phenotypes arising in zebrafish disease models due to off‐target effects of zTadA‐SpRY‐BE4max can be rectified through outcrossing.

This study provides the following recommendations for selecting the most suitable zTadA‐CBE tools based on various application scenarios in practical modeling. First, assess whether there is an NGG PAM downstream of the target cytosine. If an NGG PAM is present, prioritize the selection of two tools based on SpCas9. Depending on the specific position of the target cytosine relative to the NGG PAM, utilize either zTadA‐BE4max with the C4–C8 editing window or zTadA‐BEmv with the C9–C16 editing window. Conversely, if there is no NGG PAM downstream of the target cytosine, opt for modeling with zTadA‐SpRY‐BE4max, ensuring that the target cytosine falls within the C4–C8 window and selecting an NRN PAM whenever feasible.

In conclusion, the first zTadA‐CBE toolkit based on the TadA8e variant, developed in this study offers a potent new instrument for accurately and efficiently modeling zebrafish SNV‐related genetic diseases. This toolkit serves as a bridge between ABE and CBE methodologies in zebrafish, showcasing extensive potential applications due to its precision, effectiveness, and versatility.

## 4 Experimental Section

### Zebrafish Maintenance

Wild‐type AB zebrafish embryos were incubated at 28.5 °C. Pairs were randomly selected from AB male and female fish lines (12–15 months), maintained at an aquaculture density of 30 fish per 3‐liter tank. All animal experiments were conducted in accordance with relevant regulations and have been approved by the University Animal Care and Use Committee of South China Normal University under the approval number SCNU‐BRR‐2021‐021.

### Plasmid Construction and mRNA Generation

The pT3Ts‐zSpRY‐ABE8e plasmid^[^ ^37^ ^]^ has been modified to create the zTadA‐BE4maxes by replacing the SpRYCas9(D10A) with a synthesized fragment from zAncBE4max containing codon‐optimized zebrafish SpCas9(D10A), two UGI copies, and essential linkers. Specific mutations N46L, N46L/V82S/Q154R, N46L/E27H/I49K, and N46L/E27H/I49K/V82S/Q154R were then respectively introduced into the TadA8e deaminase domain to generate the plasmids pT3Ts‐zTadA‐BE4max‐a, pT3Ts‐zTadA‐BE4max‐b, pT3Ts‐zTadA‐BE4max‐c, and pT3Ts‐zTadA‐BE4max. The plasmid pT3Ts‐zTadA‐SpRY‐BE4max was engineered by substituting the SpCas9 (D10A) domain of pT3Ts‐zTadA‐BE4max with the codon‐optimized zebrafish SpRYCas9 (D10A), which was amplified from the zSpRY‐ABE8e plasmid. The zTadA‐BEmv was constructed based on the pT3Ts‐zTadA‐BE4max. The N‐terminal TadA deaminase domain of pT3Ts‐zTadA‐BE4max was first deleted, and then the HNH domain of SpCas9 (amino acid segment S793‐R919) was replaced with the synthetic segment containing the codon‐optimized wild‐type TadA and TadA8e variants (N46L/E27H/I49K/V82S/Q154R) to generate pT3Ts‐zTadA‐BEmv plasmid. The PCR amplification above were performed using the Vazyme High‐Fidelity DNA Polymerase 2x Phanta Max Master Mix. To introduce desired mutations, fragments and deletions, the Vazyme Mut Express II Fast Mutagenesis Kit V2 was used for infusion cloning. The primers for plasmid construction are listed in Data S1 (Supporting Information).

For mRNA synthesis, plasmids were linearized with XbaI, then were purified as a template for in vitro transcription which was performed with the T3 mMESSAGE mMACHINE kit from Ambion. Finally, the mRNA was purified using the RNA Clean Kit from TIANGEN Biotech (Beijing) Co., Ltd.

### gRNA Generation

Chemically synthesized by GenScript EasyEdit sgRNA Synthesis Services, each gRNA featured MS modifications at both ends. These custom gRNAs were prepared in a stock solution with a concentration of 1000 ng µl^−1^ and stored at −80 °C. The target sequences are detailed in Data S2 (Supporting Information).

### Microinjection and Base Editing Analysis

At the single‐cell stage, zebrafish embryos were injected with a 2 nl solution comprising 400 ng µl^−1^ CBE mRNA and 200 ng µl^−1^ gRNA, as previously described.^[^ ^49^ ^]^

For base editing analysis, three pools of six randomly selected injected embryos were collected. Genomic DNA was extracted using alkaline lysis, followed by PCR amplification with primers flanking each sgRNA target site at ≈100 bp upstream and downstream. PCR products were Sanger sequenced and analyzed with the program version 1.0.10.^[^ ^50^ ^]^ A list of PCR amplification and Sanger sequencing primers is provided in Data S3 (Supporting Information).

### Alcian Blue Cartilage Staining

Zebrafish embryos (5 dpf) were fixed in 4% paraformaldehyde in 1× diethyl pyrocarbonate (DEPC)‐phosphate‐buffered saline (PBS) overnight at 4 °C. Following fixation, embryos were dehydrated by gradient ethanol solution and stained with 0.15% Alcian Blue (Shanghai Sangon) in 75% acidic ethanol for 90 min at room temperature, followed by thorough washing with PBS. Subsequently, the embryos were hydrated by gradient ethanol solution and treated with 0.25% trypsin at 37 °C for 4 h, followed by incubation in a 1% KOH solution in 3% H_2_O_2_ for 2 h. After a gradient ethanol dehydration step, the stained embryos were stored in 70% glycerol at 4 °C.

### Whole Mount Immunofluorescence

Fix embryos in 4% PFA overnight at 4 °C. Remove the PFA, prepare a 1:1 mixture of PBST and CUBIC‐I solution, add it to the EP tube, and incubate at 37 °C in the dark for 24 h. Wash the embryos with PBST five times, allowing 10 min for each wash. Block the embryos in 5% FBS (dissolved in PBST) for at least 4 h at room temperature. After blocking, remove the blocking buffer and incubate the embryos with the primary antibody (1:500, diluted in blocking buffer) overnight at 4 °C. Wash again with PBST five times for 10 min each time. Next, block the embryos in 5% FBS (dissolved in PBST) for at least 20 min at room temperature. After blocking, remove the mixture in the EP tube and incubate with the secondary antibody (1:500, diluted in blocking buffer) and DAPI (1:500, diluted in blocking buffer) overnight at 4 °C. Wash the embryos with PBST five times for 10 min each time. Finally, remove the PBST, and add CUBIC‐II solution to the EP tube, incubating overnight at 4 °C.

Primary antibody: Rabbit anti‐L‐plastin (Ab3‐lcp1),^[^ ^51^ ^]^ which specifically marks macrophages, is a kind gift from Prof. Zilong Wen at Southern University of Science and Technology.

Secondary antibody: Donkey anti‐Rabbit‐488 nm from Jackson ImmunoResearch Inc. (#711‐547‐003).

### Image Acquisition

For albino zebrafish mutants, 3 dpf embryos were anesthetized with a 0.03% Tricaine solution (Sigma–Aldrich) and gently mounted in 4% methylcellulose. Imaging was performed using an SZX10 Stereoscope camera (OLYMPUS). For ARS zebrafish mutants, embryos were mounted in 70% glycerol after Alcian Blue staining. Imaging was performed using an XM10 digital camera (OLYMPUS). Subsequent post‐capture adjustments and enhancements were carried out using Adobe Illustrator and Adobe Photoshop. For the *fms^ts^* series zebrafish, embryos at 36 hpf were treated with immunofluorescence, and imaging was performed using an LSM 900 laser confocal microscope (Zeiss).

### Off‐Target Analysis

For each gRNA, CRISPOR (Version 4.99) was applied to predict potential off‐target sites.^[^ ^44^ ^]^ Based on the specificity scores calculated by CRISPOR (Data S4, Supporting Information), the top three available sites were identified as the most probable off‐target candidates. Their potential impact was subsequently assessed through NGS analysis.

### Next‐Generation Sequencing (NGS) and Analysis

For NGS library construction, PCR was conducted to amplify genomic DNA segments at targeted and off‐target sites, with sequence lengths varying from 100 to 280 bp. Sequencing was performed using an Illumina MiSeq instrument in PE150 mode through a commercial service provided by Biomarker Technologies. The obtained sequencing data underwent CRISPResso2^[^ ^52^ ^]^ analysis to evaluate the efficiency of genome editing and the occurrence of indels. The primers utilized for NGS are detailed in Data S5 (Supporting Information). The raw NGS data have been uploaded to the National Center for Biotechnology Information Sequence Read Archive under the following accession numbers: PRJNA1147639, PRJNA1156393, and PRJNA1148048.

### Statistics

Experiments were conducted independently three times to ensure data robustness. Statistical analysis was performed using GraphPad Prism 9, with results presented as mean ± standard deviation (SD). A two‐tailed unpaired t‐test was used to evaluate significant differences between groups, setting the significance threshold at *P* < 0.05. Significance is indicated by asterisks: ^*^ for *P* < 0.05, ^**^ for *P* < 0.01, ^***^ for *P* < 0.001, and ^****^ for *P* < 0.0001.

## Conflict of Interest

Y.L., JF.F., S.Z., and Y.L. have a pending patent application on the presented framework. All other authors declare they have no competing interests.

## Author Contributions

Y.L. and J.F.F. conceived the project. S.Z., Y.L., and J.F.F. designed the experiments. S.Z., Y.L. X.X., and J.X. did most of the experiments and analyzed the data. H.M., Y.Z, X.Y., Z.C., G.P., and W.L. contributed to the experimental works and data analysis. Y.L. wrote the manuscript draft. Y.L. and JF.F. supervised the work and were responsible for the funding acquisition. All authors read and approved the manuscript.

## Supporting information



Supporting Information

Supplemental Table 1

## Data Availability

The data that support the findings of this study are openly available in [National Center for Biotechnology Information Sequence Read Archive] at [https://www.ncbi.nlm.nih.gov/sra], reference number [1147639].
