# Supplementary material for: Sequence Context‐Agnostic TadA‐Derived Cytosine Base Editors for Genome‐Wide Editing in Zebrafish
Source: Adv Sci (Weinh). 2025 Feb 17;12(14):2411478. doi: 10.1002/advs.202411478 (PMC11984895; doi:10.1002/advs.202411478)
Supplement: Supplementary file 1 — Supporting Information [file ADVS-12-2411478-s001.pdf]

ADVANCED SCIENCE
Open Access

Supporting Information

for *Adv. Sci.*, DOI 10.1002/advs.202411478

Sequence Context-Agnostic TadA-Derived Cytosine Base Editors for Genome-Wide Editing in Zebrafish

Shaohui Zheng, Yang Liu, Xinxin Xia, Jiawang Xiao, Hui Ma, Xuanyao Yuan, Yan Zhang, Zixi Chen, Guangcong Peng, Wenyuan Li, Ji-Feng Fei and Yanmei Liu**

Supplementary Materials for

Sequence context-agnostic TadA-derived cytosine base editors for genome-wide editing in zebrafish

Shaohui Zheng[1,2#], Yang Liu[1,2#], Xinxin Xia[1,2#], Jiawang Xiao[1,2#], Hui Ma[3], Xuanyao Yuan[1,2], Yan Zhang[1,2], Zixi Chen[1,2], Guangcong Peng[1,2], Wenyuan Li[4], Ji-Feng Fei[3,5,6*], Yanmei Liu[1,2*]

This PDF file includes:

Figure S1 to S8
Table S1
Supplementary Data 1 to 5

Figure S1.

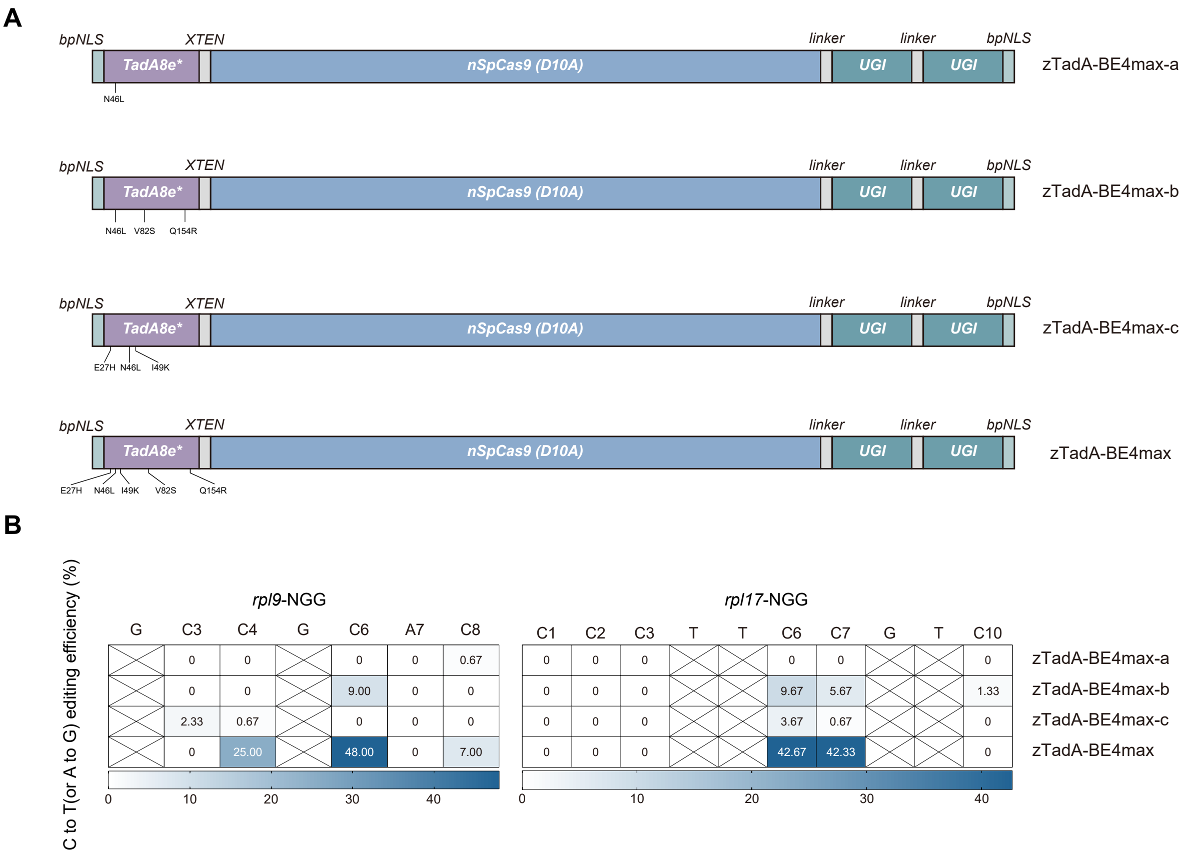

	rpl9 G	rpl9 C3	rpl9 C4	rpl9 G	rpl9 C6	rpl9 A7	rpl9 C8	rpl17 C1	rpl17 C2	rpl17 C3	rpl17 T	rpl17 T	rpl17 C6	rpl17 C7	rpl17 G	rpl17 T	rpl17 C10
zTadA-BE4max-a		0	0		0	0	0.67	0	0	0			0	0			0
zTadA-BE4max-b		0	0		9.00	0	0	0	0	0			9.67	5.67			1.33
zTadA-BE4max-c		2.33	0.67		0	0	0	0	0	0			3.67	0.67			0
zTadA-BE4max		0	25.00		48.00	0	7.00	0	0	0			42.67	42.33			0

Figure S1: Base editing efficiency mediated by zTadA-BE4maxes. (**A**) Schematic of the mRNA construct for zTadA-BE4maxes. (**B**) Comparison of editing efficiency targeting two loci with NGG PAM among four variations of zTadA-BE4maxes.

Figure S2.

49% identity and 67% similar in 99% overlap

```
HPS5     1 MAFVPVIP-ESYSHVLAEFESLDPLLSALRLDSSRLKCTSIAVSRKWLALGSSGGGLHLIQKEGWKHRLFLSHREGAIS   78
hps5     1 --MIPVVPVESCTHVLAEFDCLDPLLSALRLDSGRIKCTCLSVSRKWLALGTSAGGLHLIQRDGWKQKLILTHKEGSIT   77

HPS5    79 QVACCLHDDDYVAVATSQGLVVVWELNQERRGKPEQMYVSSEHKGRRVTALCWDTAILRVFVGDHAGKVSAIKLNTSKQ  157
hps5    78 QVSCCPHDEDFIAVATSQGLVVVWELHLERRGRPERASVSWEHRGVTVTSLCWDTVALRVFAGDMGGKVSCVRAGSSKL  156

HPS5   158 AKAAAAFVMFPVQTITTVDSCVVQLDYLDGRLLISSLTRSFLCDTEREKFWKIGNKERDGEYGACFFP-------GRCS  229
hps5   157 GK-GSAFVIFPVQTVTTVDSRVVQLGYTDGHLVISSLSRCYLCDTEREKFWRVGNKERDGEFGACFLTQGLAGQRGQLV  234

HPS5   230 GGQQPLIYCARPGSRMWEVNFDGEVISTHQFKKLLSLPPLPVITLRSEPQYDHTAGSSQSLSFPKLLHLSEHCVLTWTE  308
hps5   235 GCPAPLLFCARPGSRIWEASFSGEVLSTHQFKQLLAVPPLPLVSCKNEPHFNPTQTNPQSLAFPRLLQFGDQNLLTWTD  313

HPS5   309 RGIYIFIPQNVQVLLWSEVKDIQDVAVCRNELFCLHLNGKVSHLSLISVERCVERLLRRGLWNLAARTCCLFQNSVIAS  387
hps5   314 SAIYIFTPHSGQVLLWTEVKDVLEISVFRNDLFCLHGDGHLSHMSLVSPDRCVERLMKRENWTIAATVCCMFQHAITTS  392

HPS5   388 RARKTLTADKLEHLKSQLDHGTYNDLISQLEELILKFEPLDSACSSRRSSISSHESFSILDSGIYRIISSRRGSQSDED  466
hps5   393 KARKSLSIDRLEHLKAQLNSTSQQQLIGQLEEVISKLEPLDSACSSRRSSISSHESFNVLDCGIYRVI-SRRGSQSDDD  470

HPS5   467 SCSLHSQTLSEDERFKEFTSQQEEDLPDQCCGSHGNEDNVSHAPVMFETDKNETFLPFGIPLPFRSPSPLVSLQAVKES  545
hps5   471 ASSLANQSMLEEERLKEFSFTEEEQVDNDSASVRG------------EGDRSDLGLQF-LPLPFRSKPPRVALQAVRDS  536

HPS5   546 VSSFVRKTTEKIGTLHTSPDLKVRPELRGDEQSCEEDVSSDTCPKEEDTEEEKEVTSPPPEEDRFQELKVATAEAMTKL  624
hps5   537 VSSFMKKTTEKINTLQMNADLWPRPDLR---EGVQGEVASTASPISEESEQELNTEESSSESE-LLELRAATKKAISQI  611

HPS5   625 QDPLVLFESESLRMVLQEWLSHLEKTFAMKD-FSGVSDTDNSSMKLNQDVLLVNESKKGILDED-------NEKEKRDS  695
hps5   612 QDPMVLLDPLCLSDVLQEWAPVLERALGPEDQILPVETTNPEEKTLEEEELVSSMSCCVVVQPEISTSPAADPDESATH  690

HPS5   696 LGNEESVDKTACEC--VRS---PRESLDDLFQICSPCAIASGLRNDLAELTTLCLELNVLNSKIKSTSGHVDHTLQQYS  769
hps5   691 TEEEDFRESTPCSIAPVRAQFPPLANHVELIQLFSPKPLPPDLQADLSLLACLYLEMGC------PGRGGMES------  757

HPS5   770 PEILACQFLKKYFFLLNLKRAKESIKLSYSNSPSVWDTFIEGLKEMASSNPVYMEMEKGDLPTRLKLLDDEVPFDSPLL  848
hps5   758 ----VCVFLRRFFFLLDQERVRRMCMLRYRENREVLKAYIAGMLEFTQASKVVEVIQKGDLLKSLRSLRELQPWNAPLL  832

HPS5   849 VVYATRLYEKFGESALRSLIKFFPSILPSDIIQLCHHHPAEFLAYLDSLVKSRPEDQRSSFLESLLQPESLRLDWLLLA  927
hps5   833 LSHLYRLYEKHGEVAVRAYPQFYPTILPSDIMAMA--LPSHFLPYLDNLVQSRAEQQRLSFLGSLLQPETLRQDWLELA  909

HPS5   928 VSLDAPPSTSTMDDEGYPRPHSHLLSWGYSQLILHLIKLPADFITKEKMTDICRSCGFWPGYLILCLELERRREAFTNI 1006
hps5   910 LSHDAPQREDTLTHDGQPRWHSHFFSWGYGRLLSLLIRLPADLASKQKMLDMCKAHGYWMGYLYLCRELQRRAEAFSAI  988

HPS5  1007 VYLNDMSLMEGDNGWIPETVEEWKLLLHLIQ----SKSTRPAPQESLNGS--------------LSDGPSPINV----E 1063
hps5   989 CRLDDMTLLEGDDGIVPQSLDEWVLLLQLSQQISASDESSLTSTKNSNGSCLDDANSNGDCSSGLSNGSTDWSIQVSPE 1067

HPS5  1064 NVALLLAKAMGPDRAWSLLQECGLALELSEKFTRTCDILRIAEKRQRALIQSMLEKCDRFLWSQQA             1129
hps5  1068 NIILRLVRVFGPDRALTALQEHGIPVDHSSRSTLVCDLLRMAEKRQRALIQSMLERCDRFLWSQHA             1133
```

Figure S2: Amino acid sequence alignment results of human HPS5 protein and zebrafish hps5 protein. The red box indicates the targeted amino acids.

Figure S3.

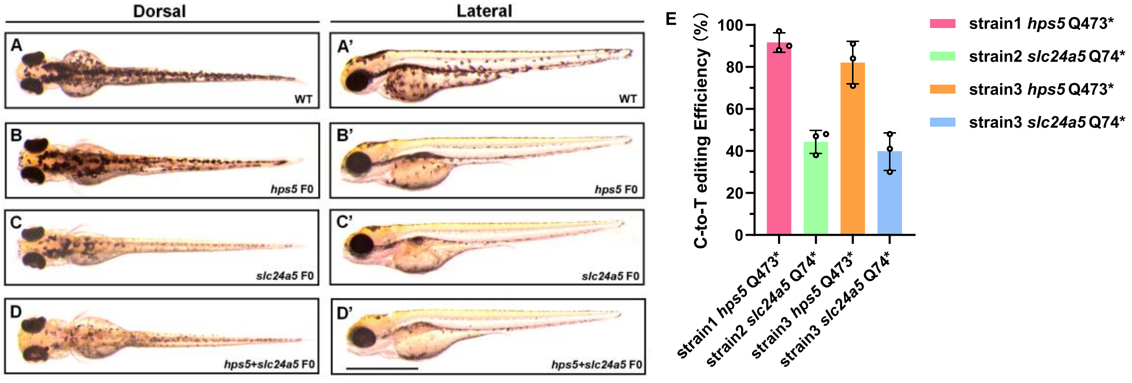

Figure S3: Modeling complex polymorphic diseases using zTad-BE4max.
(A, A'-D, D') Dorsal (left) and lateral (right) views of 4 dpf wild-type (WT) zebrafish (A, A'), strains1 (*hps5*^{Q473*}) (B, B'), strain2 (*slc24a5*^{Q74*}) (C, C'), and strain3 (*hps5*^{Q473*}*slc24a5*^{Q74*}) (D, D'). (E) Editing efficiency of the *hps5*^{Q473*} and *slc24a5*^{Q74*} sites in each strain. Scale bar: 1mm.

Figure S4.

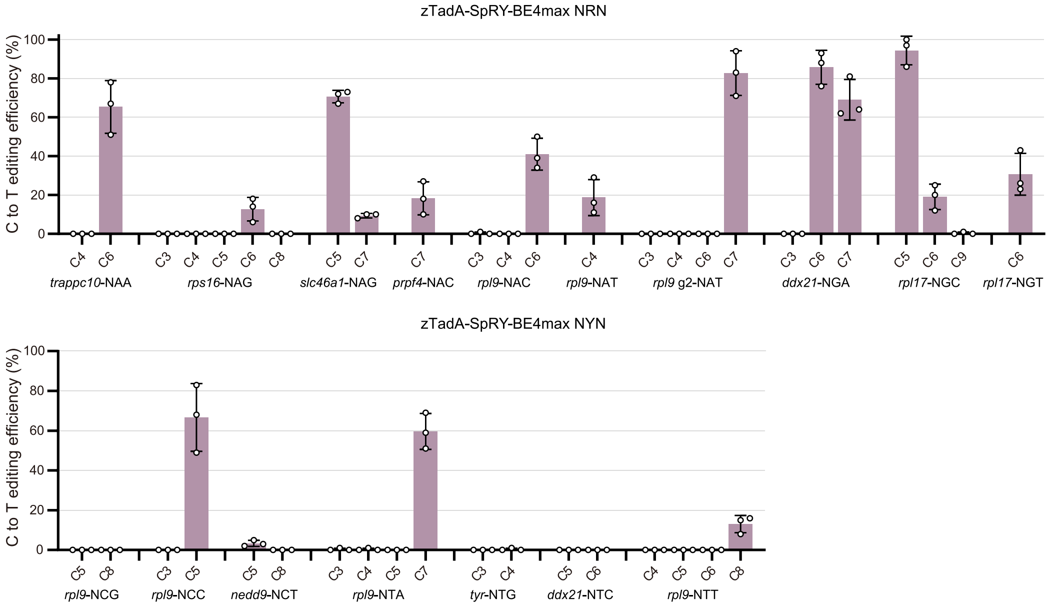

Figure S4: The editing efficiency of zTadA-SpRY-BE4max using seventeen gRNAs targeting NRN PAMs (top) and NYN PAMs (bottom).

Figure S5.

85% identity and 89% similar in 100% overlap

```
PITX2    1 METNCRKLVSACVQLEKDKSQQGKNEDVGAEDPSKKKRQRRQRTHFTSQQLQELEATFQRNRYPDMSTREEIAVWTNLTEA  81
pitx2    1 MDSHCRKLASTCAQLEKEKG-QSKNED-SNDDPSKKKRQRRQRTHFTSQQLQELEATFQRNRYPDMSTREEIAVWTNLTEA  79

PITX2   82 RVRVWFKNRRAKWRKRERNQQAELCKNGFGPQFNGLMQPYDDMYPGYSYNNWAAKGLTSASLSTKSFPFFNSMNVNPLSSQ 162
pitx2   80 RVRVWFKNRRAKWRKRERNQQAELCKNGFGPQFNGLMQPYDDMYPSYTYNNWAAKGLTSASLSTKSFPFFNSMNVNPLSSQ 160

PITX2  163 SMFSPPNSISSMSMSSSMVPSAVTGVPGSSLNSLNNLNNLSSPSLNSAVPTPACPYAPPTPPYVYRDTCNSSLASLRLKAK 243
pitx2  161 TMFSPPNSISSMSMSSSMVPSAVTGVPGSSLNSLNNLNNLSNPSLNSGVPTPACPYAPPTPPYVYRDTCNSSLASLRLKAK 241

PITX2  244 QHSSFGYASVQNPASNLSACQYAVDRPV                                                     271
pitx2  242 QHSSFGYASVQNPASNLSACQYAVDRPV                                                     269
```

Figure S5: Amino acid sequence alignment results of human PITX2 protein and zebrafish pitx2 protein. The red box indicates the targeted amino acids.

Figure S6.

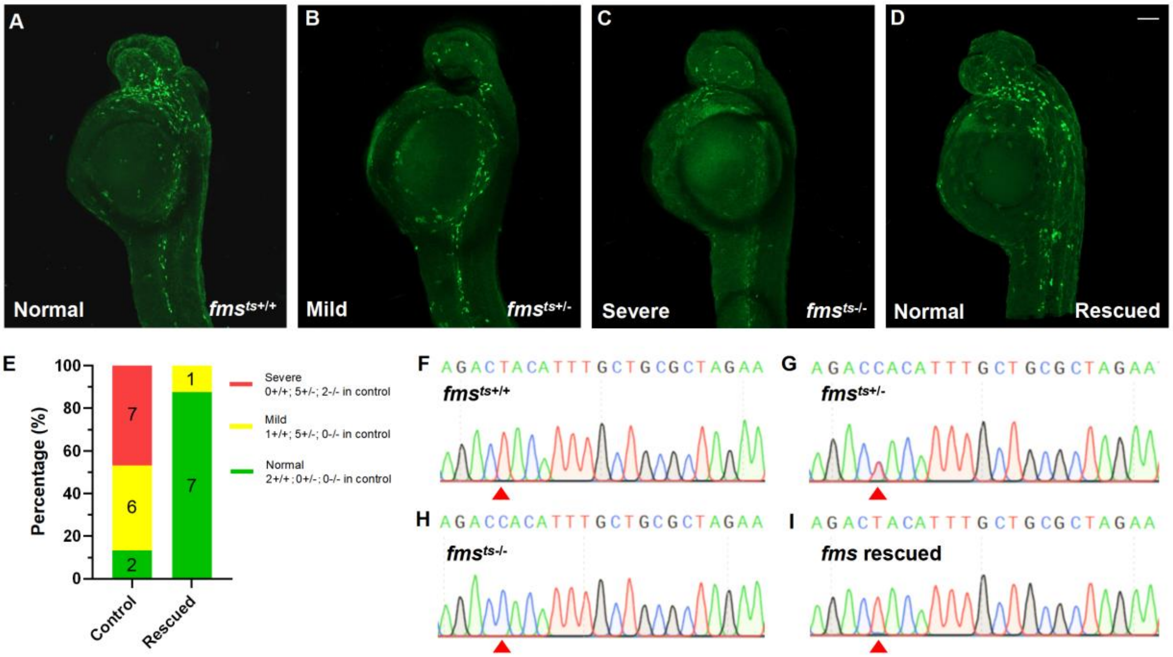

Figure S6: Utilizing zTad-SpRY-BE4max to rescue the phenotype of decreased macrophage numbers in fms^{ts}. Images of 36 hpf *fms*^{ts} zebrafish with normal macrophage numbers (*fms*$^{ts+/+}$) (A), mild decreased macrophage numbers (*fms*$^{ts+/-}$) (B), severe decreased macrophage numbers (*fms*$^{ts-/-}$) and *fms*^{ts} embryos rescued by injection of zTad-SpRY-BE4max mRNA and *fms* rescue gRNA (D). Bright green dots indicate the macrophages. (E) The proportions of the fish with normal macrophages numbers, mild decreased or severe decreased macrophage numbers in both the control and the injection group. (F-I) Sanger Sequencing results of *fms*$^{ts+/+}$ (F), *fms*$^{ts+/-}$ (G), *fms*$^{ts-/-}$ (H) and rescued *fms* (I). Scale bar: 100μm.

Figure S7.

53% identity and 69% similar in 100% overlap

```
HPS1    1 MKCVLVATEGAEVLFYWTDQEFEESLRLKFGQSENEEEELPALEDQLSTLLAPVIISSMTMLEKLSDTYTCFSTENGNFL  80
hps1    1 MKCLLVANESAEVLFYWTDSEFEQRLQEQYGVSQEEGEGLPAFEDSINTLFAPIIISCSTMVDRLGDNYTSFSTEN-NHI  79

HPS1   81 YVLHLFGECLFIAINGDHTESEGDLRRKLYVLKYLFEVHFGLVTVDGHLIRKELRPPDLAQRVQLWEHFQSLLWTYSRLR 160
hps1   80 YVLHQFDECLYIAVNGDGEETEEDLKRKIYVMKKLTEILFGMVTLSGPLLRKELRPQDTEQRNRLWKKLRSLLETYSRLR 159

HPS1  161 EQEQCFAVEALERLIHPQLCELCIEALERHVIQAVNTSPERGGEEALHAFLLVHSKLLAFYSSHSASSLRPADLLALILL 240
hps1  160 ENDQSFLVEAVERLIHPTLCEQCIEFLERRLVQQINSSMDRAGEEVLHAFILVHTKQLAFYSSRNASNLNPSDLLALIIL 239

HPS1  241 VQDLYPSESTAEDDIQPSPRRARSSQNIPVQQAWSPHSTGPTGGSSAETETDSFSLPEEYFTPAPSPGDQSSGSTIWLEG 320
hps1  240 VQDLYPSKIDLDD---------------------------------TPEELENSTVPDVFYTPEPSPPERESVTP---RK 283

HPS1  321 GTPPM-----DALQIAEDTLQTLVPHCPVPSGPRRIFLDANVKESYCPLVPHTMYCLPLWQGINLVLLTRSPSAPLALVL 395
hps1  284 DSPPVFQFVDPDIQMAEDSLQTLEVSTPDPSSPSRVFLEAKE----CPMMPHSMYCLSLWPGITLVLLTKIPNSHMAVSV 359

HPS1  396 SQLMDGFSMLEKKLKEGPEPGASLRSQPLVGDLRQRMDKFVKNRGAQEIQS-----TWLEFKAKAFSKSEPGSSWELLQA 470
hps1  360 YYFLEAFVKLEKRLGEGHEGASAMRGQSSVQEQRSKLEKFIKAWSSMEIQTLQLQNAWTDFKNKAFSRSGTGFTRDLLPS 439

HPS1  471 CGKLKRQLCAIYRLNFLTTAPSRGGPHLPQHLQDQVQRLMREKLTDWKDFLLVKSRRNITMVSYLEDFPGLVHFIYVDRT 550
hps1  440 CRNMKTQLCGVYR-QFFAAECMGSSQRLAPHLQERALNMVQEKLMDWKDFLLVKSKRNITMVSYLEEFPGLIHFIYVDRS 518

HPS1  551 TGQMVAPSLNCSQKTSSELGKGPLAAFVKTKVWSLIQLARRYLQKGYTTLLFQEGDFYCSYFLWFENDMGYKLQMIEVPV 630
hps1  519 SGQMIAPSLNVTDRTVSELGKGPLADFIKKKVWSLVATARRYLHKGYATVTLRDGDYFYCYFLWFENETGYKLEVTDIPS 598

HPS1  631 LSDDSVPIGMLGGDYYRKLLRYYSKNRPTEAVRCYELLALHLSVIPTDLLVQQAGQLARRLWEASRIPLL            700
hps1  599 CPDDTAPIGMLTFDYYRKLLRYYSKKHQNEVVKCYELLTVHLGVIPNEYILQHCSQLARKLWEPTRIPLL            668
```

Figure S7: Amino acid sequence alignment results of human HPS1 protein and zebrafish hps1 protein. The red boxes indicate the targeted amino acids.

Figure S8.

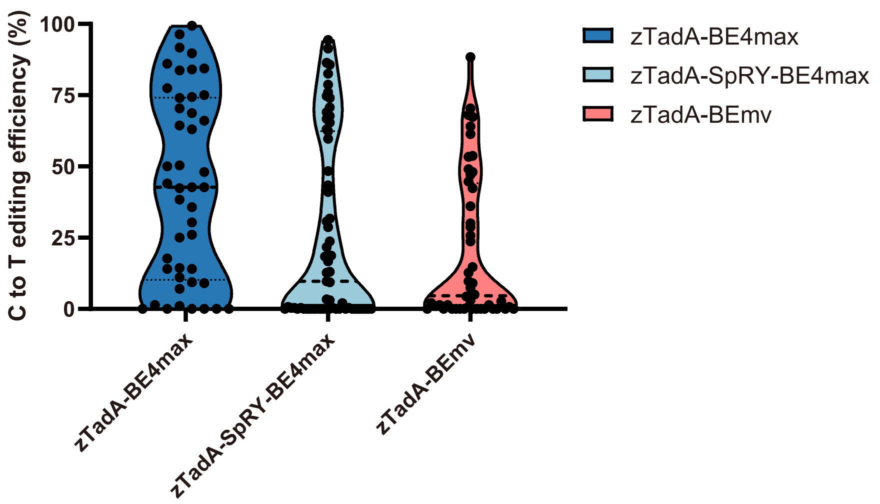

Figure S8: Summary of the editing efficiency of zTadA-CBEs. Data from all tested loci, and dotted line in the middle indicates median.

Table S1.

Base editor	Gene locus	germline targeting efficiency	germline transmission efficiency
zTadA-BE4max	*hps5* Q531*	75.00% (6/8)	6#,64.00% (16/25)
zTadA-SpRY-BE4max	*hps1*-NGC	100.00% (4/4)	3#,92.00% (23/25)
zTadA-BEmv	*hps1* Q140*	85.71% (6/7)	5#,100.00% (25/25)

Table S1. Germline targeting efficiency and Germline transmission rate of zTadA-CBEs. Germline targeting efficiency quantifies the percentage of injected F0 adult fish capable of generating mutant F1 offspring through mating with wild-type fish. The germline transmission rate reflects the proportion of heterozygous F1 offspring resulting from the crossbreeding of founder F0 fish with wild-type fish.

Supplementary Data 1. Primers for plasmid construction in this study.

Products	Sequence of the primer (5’-3’)
zAncBE4max backbone	F: GGCGGCAGTGATAAGAAGTATAGCATCGGCCTGGC
	R: CCTTTAGTGAGGGTTAATTTCGAGCTTGGCGTAA
T3-TadA8e	F: AATTAACCCTCACTAAAGGGAACAAAAGCTGGAG
	R: TACTTCTTATCACTGCCGCCAGAGGAGCCTCCACTG
N46L	F: GAGGGATGGCTGCGCGCAATTGGACTGCATGACCC
	R: ATTGCGCGCAGCCATCCCTCGCCTATAACCCTGTTATTC
V82S	F: CACTGTATAGCACCTTCGAGCCTTGTGTGATGTGC
	R: CGAAGGTGCTATACAGTGTGGCGTCAATGAGTCTA
Q154R	F: AATGCCAAGACGGGTTTTCAACGCTCAGAAGAAAG
	R: TGAAAACCCGTCTTGGCATTCTGTAGAAATCGCACAG
E27H	F: GAGATGAGAGGCATGTTCCTGTGGGCGCAG
	R: AGGAACATGCCTCTCATCTCTAGCCCTCTTTGCG
I49K	F: GGATGGCTGCGCGCAAAAGGACTGCATGACCCAAC
	R: CCTTTTGCGCGCAGCCATCCCTCGCCTATAACCCTG
zTadA-BE4max backbone	F: CGAAACAAGGATTGACCTGTCTCAGCTGGGTGGC
	R: AGCCAGGCCGATGCTATACTTCTTATCACTGCC
SpRY-nCas9	F: AGTATAGCATCGGCCTGGCTATTGGAACTAACTCCG
	R: ACAGGTCAATCCTTGTTTCGTACAGGCCGG
zTadA-BE4max remove deaminase	F: GGGAAGCATGGATAAGAAGTATAGCATCGGCCTGGCTATTGG
	R: ACTTCTTATCCATGCTTCCCACCTTTCTCTTTTTCTTGGG
zTadA-BEmv backbone	F: TTCCATTAATAGCGGAGGAAGGCAGCTGGTGGAAACACGCCAGATCACTAAAC
	R: CGACTTCGCTGGATCCTCCGCTACCCAGCTCCTTAATG
TadA deaminases	F: CGGAGGATCCAGCGAAGTCGAGTTTTCACACGAATAC
	R: TTCCTCCGCTATTAATGGAAGACTGGGCTTTCTTCTGAGCG

Supplementary Data 2. All target loci used in this study.

sgRNA name	Target loci (N20 + PAM)
pitx2 g1	GACGGGCAAAATGGAGAAAAAGG
rpl9- NGG	GGCCGCACAGTTACCGTGAAGGG
rpl17 -NGG	CCCTTCCGTCGCTACAACGGGGG
rps16 -NGG	CGCTGCGAGTCCAAGAAGTTTGG
jag1a	TTCCAGTGTTTGTGCCCGCCTGG
tyr TCGC	GCAATCCCGGGGACCACGACCGG
tyr g3	TGAAGCTCAGGTTTGCCCGTCGG
slc22a7a	GGCATCCCACGTAATTCAGATGG
kmt2d g1	TAGGCAACAAGTATCTCTACTGG
slc24a5 (Q74*)	GTGCAGGAGAGGAAAGATGGAGG
hps5 Q473* (g1)	CAACCAATCAATGCTCGAGGAGG
oca2	CCGACCGGGAGAGCCCCTGGAGG
rpl9 g2	CCTTTCCCCCTTAATCGGCGAGG
rpl9 g3	ACATCCGCCGTGTCCGCATGAGG
rpl9 g4	AACACCACGGGGTCCCTTCACGG
rpl9 g5	TGCAGTCACGGTGTCCCTCAAGG
hps5 Q531* sgRNA	CCCTGCAGGCTGTTCGAGACAGG
rpl9 -NAA	AAGGGCCGCACAGTTACCGTGAA
trappc10 -NAA	TGACTCAGCAGCTGCCCAGAGAA
rpl9 g2-NAG	AGGGCCGCACAGTTACCGTGAAG
rps16 -NAG	ATCCCCGCCGCTGCGAGTCCAAG
slc46a1 -NAG	CGGTCACAGACAACGGGCACGAG
rpl9 -NAC	TCCCTCAAGGGCCGCACAGTTAC
rpl9 -NAT	AGACAGTGGACATCCCTGACAAT
rpl9 g2-NAT	CTCCGCCGGGAGTTCAACCA CAT
slc46a1 -NAT	GACTCAGAGTCCTCCGCGAGAAT
ddx21 -NGA	AACATCCAGCATGCCGTCAAAGA
rpl9 g2-NGA	GCCGCACAGTTACCGTGAAGGGA
rpl17 -NGC	ACATCCTTCAGGTACTTGTTAGC
pitx2 -NGC	AGCCCAGTTGTTGTACGTATAGC
hps1 -NGC	GTCCAGTAGAACAGAACCTCTGC
rpl17 -NGT	GCTAACAAGTACCTGAAGGATGT
ddx21 -NCA	CAGCCGCTCCTCAACATCCAGCA
rpl9 -NCC	CGCACAGTTACCGTGAAGGGACC
rpl9 -NTA	GTCCCTCAAGGGCCGCACAGTTA
rpl17 -NTA	CACATCCGCAAGGCTAACAAGTA
rpl9 -NTT	TGTCCCTCAAGGGCCGCACAGTT
pitx2 Q48* sgRNA (g4)	CCAGCAGTTACAGGAACTGGAGG
ilf3b	GAACGACGACCGCCATGTGATGG
twist2 g1	GCTCCAGAACCAGCGCGTCCTGG
pitx2 g3	TCAAGAATCGACGGGCAAAATGG
hps1 Q140* sgRNA	AAGACACAGAGCAGAGGAACCGG
dnah10	CAGAGGCGGAAGTCTGGATGTGG
gja1b g1	AACAGCAGTGGAATCGGCCTGGG
gja1b g2	CAGAAGCGCACGTGCGAGATGGG
kif14	AGCCCAGACTCATCTAGTGAAGG
pitx2 g5	ATAGCGATTCCTCTGAAAAGTGG
mib1	GGTCAACATTGATTTGGACCTGG
slc46a1 -NCA	ACGGTCACTCCATGAGCCCAGCA
shroom4 -NTG	CCGTTCAGCTGCACATGGATGTG
prpf4 -NAC	CTATAGCAGACATTGAGGGACAC
fms	AGACTACATTTGCTGCGCTAGAA
rpl9 -NCG	TGTGCGGCCCTTGAGGGACACCG
nedd9 -NCT	CCATCAGCAGCCAGAGCTCCTCT
tyr- NTG	GTCCGATGGGGGCGTTGGCGGTG
ddx21 -NTC	ACGGCCTGCAGCTGCTCAAGGTC
kmt2d 2099	CGTGTGGCCTCATAACCGACTGG
tuba1a 383	CACAGCTATTGCTGAGGCCTGGG
ptpn11	CAAACTTCTCTCCACCATACAGG
lrp5 g3	CTGATGGAATTGCTGTGGATTGG
lrp5 g4	AACCGTGGTGCAGGGACACCTGG
twist2 g2	GCCGCTCGCGTACGTTCGCCAGG

Supplementary Data 3. Primers for PCR amplification and Sanger sequencing in this study.

Gene	sgRNA name	Sequence of the primer (5'-3')	Sequencing primer set
rpl9	*rpl9* -NGG/g4/g5 and all non-NGG PAM sgRNAs	F: CCATTGAGGCGACTCGAGAA	√
		R: TGCATTTTAACAACTCCAGCAAGA	
	rpl9 g2	F: CGCAAAACGTACTGGTTACATTTA	
		R: GCTACGGCCTTTGAGTGCCGCT	√
	rpl9 g3	F: GTTTCCCAGGGCTTCAGGTAC	√
		R: CTCACCTGAGTTTGACACCAGC	
rpl17	all *rpl17* sgRNAs	F: GCCCTAGTGCCAGTGAAGTT	√
		R: AGCATTTCATGTTGTGCTTATGAC	
rps16	all *rpl16* sgRNAs	F: CAGCAAATTAAGAAATGTAC	√
		R: GAGATTTTAACGGTAGGATT	
pitx2	*pitx2* g1/g3	F: CCTAACTGAGTTTGACACCACC	√
		R: GGAAAGCTTTTGGTAGACAGAGAG	
	pitx2 Q48* sgRNA (g4)	F: TGCATTACAGAGAAAGAAAAGGGACA	√
		R: AATTTTATTTATCAAACTTACTCGGA	
	pitx2 -NGC	F: GTTTGACACCACCACCCATATC	
		R: TGGTACACCGGTGACAGCAG	√
	pitx2 g5	F: TGCATTACAGAGAAAGAAAAGGGACA	√
		R: AATTTTATTTATCAAACTTACTCGGA	
kmt2d	*kmt2d* g1	F:AGACGTTCTGTCGGAATTCC	√
		R:CTAAAGAATGCTACTTAACTG	
	kmt2d 2099	F: TGATCGCATTCACTTGTTCCGTGT	
		R: TCTGGAGTGGAGTCTCTGAAGTGC	√
slc22a7a	*slc22a7a*	F: GTTTGGATGGCAGGTTCTCAG	√
		R: CATGGCACAGACACATTTGAC	
gja1b	*gja1b* g1/g2	F: GCTAGAACTCCCTCAAGATGGGTG	√
		R: TTTACGGTTGAGTTTCTCCTCC	
dnah10	*dnah10*	F: TTGATGCTACAAAACTGTCACCTGT	
		R: AAGCATGAGCTGACGTGAGAAATAC	√
kif14	*kif14*	F: CACTTCGAACAAAGCTGTGC	√
		R:CAAGCACCTGAATGAGTAACTGC	
oca2	*oca2*	F: ACAGATATTGCCTAGGGATGAC	√
		R: GACAGTGGCAGCATACTATTTG	
mib1	*mib1*	F: CTGGTTCAGCTGAATTTTCCTAG	√
		R: CTGTGGTGGTGAGCGTCTCGAAC	
hps5	*hps5* Q531* sgRNA	F: ACCTTCAAAACATGAGCGAGC	√
		R: GTGTGTCGCACAATATGAGGG	
	hps5 g1(Q473*)	F: CTCTACTCTGCACAGGAGAGC	√
		R: GTCTCTCTCAGTCTCACCGTTA	
jag1a	*jag1a*	F:GCTTCAGTCAGTATGTGATTG	√
		R:CCCAAATGATGCATTGTAC	
nedd9	*nedd9* -NCT	F: CGCCCCTGGCAACCGCCTC	√
		R: TACTACATTTGATCTTAGTG	
trappc10	*trappc10* -NAA	F:TCCAGTCATAGACTGAGACGC	√
		R:TGTTGCGCAGATGCGGGTGATC	
ilf3b	*ilf3b*	F: CACACTCATAAGTTTGCTACAAGG	√
		R: CTTCAGATCTACTCACGTCTCC	
twist2	*twist2* g1/g2	F: CTGGACAGACAGCAGAAAAGGTTC	
		R: ACTTGCTGAAGTCTGGCATCTCTC	√
slc24a5	*slc24a5* (Q74*)	F: CATCCAGCATGTCCTCATAAAGAG	√
		R: GTGTGTAGAGAATGCGAGTGAGTG	
tyr	*tyr* TCGC/g3	F: GGCTTTAAAGTGGACGCTGTC	
		R: TCAGCCTGAAAGTTACAACCTCC	√
	tyr -NTG	F: CTCTAACAACTGCTTTCCAT	√
		R: AACAACGGCCAGAACCTATAATAGT	
slc46a1	*slc46a1* -NAG/NAT	F:CATGGAGGATTCAGACACCAG	
		R:CTGCGTGCACAGAGGAGCCTGC	√
	slc46a1 -NCA	F: GAAGTGGAGACTCTGACTGC	√
		R: GCATTGAAGTCCCCTGAGAG	
hps1	*hps1* -NGC	F: GGCAAACTCGCTTTAGAAGTCC	√
		R: ACATACTCCTTCTCCTTCCTCC	
	hps1 Q140* sgRNA	F: AGTGGCCGATGAGTGTAAGG	√
		R: ACGCACCTCCACTAAAAAGC	
ddx21	*ddx21* -NCA/NGA/NTC	F:AGCTATGTACTGCAGCTCCG	√
		R:GTAGAGTTAGACCGTGTATTAT	
shroom4	*shroom4* -NTG	F: CGTGATGAGGTTGGATCGTGAC	
		R: CAAACACAGGCATGTAGAATGAG	√
prpf4	*prpf4* -NAC	F: TGGAGTTTAGACAGGTGTGTATC	√
		R: ACCAAGTTGTGCCCAGAAACC	
ptpn11	*ptpn11*	F: CTCACTAGGCGAAATGGCGCTGTC	√
		R: TGTATGTGCTCACCTCTCGGAGG	
lrp5	*lrp5* g3	F: TTGAGGGCTATACGGCGTGC	√
		R: ATGGCTCGTGGTTCGTCCAG	
	lrp5 g4	F: CTCATCCGTTCGGGTTGACC	√
		R: GCATCGACACTGGGCTCCAG	
tuba1a	*tuba1a* 383	F: GCATCAACTACCAGCCTCCC	
		R: CCAGGGCAGCCATGTCCTCC	√
fms	*fms*	F: TCGAGTTCTCTTTGTTTCTCCGAG	√
		R: AAAGCGGTAAAACCAAACTA	

Supplementary Data 4. The predicted off-target sites in this study.

Target sites	Off-target Sequence	Mismatch Position	Chromosome	Direction	Mismatches	Item number	mitOfftarget Score
hps5 Q531* sgRNA	CCCTGCAGACTTTTCGAGACAGG	........*..*........	chr4	+	2	SNP skipped	1.72036988
	CACTGTAAGCTGTTAGAGACAGG	.*...*.*......*.....	chr11	-	4	1	0.247907189
	CCTTGCATGCTGTTCGGGACAGA	..*....*........*...	chr19	+	3	SNP skipped	0.239223549
	GCCTGGAGGCGGTTAGAGACAGG	*....*....*...*.....	chr18	+	4	2	0.139991782
	CCCTGGAGGGTGGTCGGGACCGG	.....*...*..*...*...	chr10	+	4	3	0.122722595
	GACTGCAGGCTGTTCAAGCCAGG	**.............*..*.	chr23	+	4		0.090617958
	CCCAGCAGGCTTTTGGAGAGCGG	...*.......*..*....*	chr1	+	4		0.088633633
	CTCTGAAGGCTGTTCAAGAATGG	.*...*.........*...*	chr7	+	4		0.072576354
	CTCTGCTGGCTGTTGGACACTGG	.*....*.......*..*..	chr11	-	4		0.057832793
	CCCTGCGCGCTGTACGGGACAGG	......**.....*..*...	chr11	-	4		0.056971347
	CACTGCAGGCTGTTCATGATGGG	.*.............**..*	chr21	+	4		0.046184952
	CACTGCGGGCTGTGGGAGACTGG	.*....*......**.....	chr6	-	4		0.041700448
	CCCTGCAGGCTGTTTGATGCAGG	..............*..**.	chr14	+	3		0.040150713
	CCTTACAGGCTGTGCTAGACAGG	..*.*........*.*....	chr16	-	4		0.03863586
	CTCTGCAGGCCGTGTGAGACTGG	.*........*..**.....	chr18	-	4		0.03388543
pitx2 Q48* sgRNA	TCATCAGTTACAGGAACTGGTAA	*..*................	chr5	+	2	1	5.722891566
	CCAGCAGCTCCAGGAACTGGAAG	.......*.*..........	chr13	-	2	2	5.028448276
	CAAGTAGCTACAGGAACTGGAGC	.*..*..*............	chr21	-	3	3	2.543507363
	GCACCAGGTACAGGAACTGGTTA	*..*...*............	chr2	-	3		2.543507363
	AAAACAGTTACAGGAACTGGAAC	**.*................	chr16	+	3		2.31990232
	CAAGTAGTTACAGGAACTGTTAT	.*..*..............*	chr16	+	3		1.492090395
	ACCACAGATACAGGAACTGGAGC	*.**...*............	chr6	-	4		1.345833333
	CAGACAGATACAGGAACTGGATC	.***...*............	chr17	-	4		1.345833333
	TGGGCAGATACAGGAACTGGAAT	***....*............	chr10	+	4		1.345833333
	CCAAGAGTTACTGGAACTGGATT	...**......*........	chr14	-	3		1.314767932
	TCAGCAGATACAGGAATTGGAAG	*......*........*...	chr7	-	3		1.290123457
	TCAACAGTTACAGGAAATGGGTG	*..*............*...	chr15	+	3		1.290123457
	TCAACAGTTACAGGAAATGGGTG	*..*............*...	chr15	+	3		1.290123457
	CGATGAGTTTCAGGAACTGGAGG	.*.**....*..........	rUn_KN150663	-	4		1.257112069
	CACTCAGTTTCAGGAACTGGTGC	.***.....*..........	chr6	-	4		1.2395125
hps1 Q140* sgRNA	AACACACAGAGCAGAGGATCTGG	..*...............*.	chr5	-	2	1	4.759040323
	ATGACGCAGAGCAGAGGAACGGG	.*...*..............	chr23	-	2	SNP skipped	3.637658228
	AAGACACAGCGCTGAGGAACGGG	.........*..*.......	chr10	+	2	2	2.039793072
	AATCCACAGAGCAGAGGAATGGG	..**...............*	chr5	-	3	3	1.377791534
	AAGAAACTGAGCAGAGGAAAAGG	....*..*...........*	chr2	-	3		1.313930348
	CTGGGACAGAGCAGAGGAACTGG	**.**...............	chr18	-	4		1.304945055
	AACAAACAGAGCAGAGGAACAGA	..*.*...............	chr16	+	2		1.076666667
	ACAACACAGAGCAGAGGAGCAGG	.**...............*.	chr17	-	3		1.040777778
	GAGAGAGAGGGCAGAGGAACAGG	*...*.*..*..........	chr5	+	4		0.89998622
	ATTACAGAGTGCAGAGGAACTGG	.**...*..*..........	chr2	+	4		0.846587038
	AATGAACATAGCAGAGGAACAGG	..***...*...........	chr9	+	4		0.822304167
	AACACACAAAGCAGAGGAACAAG	..*.....*...........	chr7	+	2		0.806089718
	ATGAAACAAGGCAGAGGAACTGG	.*..*...**..........	chr20	-	4		0.768095474
	AATAGACGGAACAGAGGAACAGG	..*.*..*..*.........	chr11	+	4		0.7469375
	ATCACACAGCACAGAGGAACTGG	.**......**.........	chr19	+	4		0.721082663

Supplementary Data 5. Primers for NGS in this study.

Target sites	Sequence of the primer (5’-3’)
hps5 Q531* OT	F: CATCTCTGTTTCTGCTCTCCTG
	R: GAAAGTTTCCTAAATCTGTTAAACAAAG
hps5 Q531* OF1	F: GAAACTGCAACTTTATTCACAGAAG
	R: CCCCAAAAACTGCTTAGACAGT
hps5 Q531* OF2	F: CCTCCTACATATACTTGTTGTCTTTG
	R: TTAAAGGTATTGACGTGCTAGTGCT
hps5 Q531* OF3	F: GGTACCGTTGGCACTGGCAGCG
	R: CATTCTATACAAAATCCTGTTTACC
pitx2 Q48* OT	F: TGCATTACAGAGAAAGAAAAGGGACA
	R: AATTTTATTTATCAAACTTACTCGGA
pitx2 Q48* OF1	F: GAAGTACGCTGGCTACACTC
	R: CACATGATGTCTCCACTGCATG
pitx2 Q48* OF2	F: GGTTTAGTTTGGCAGGGACAC
	R: CACAGATGAGTCGAACCCAG
pitx2 Q48* OF3	F: CTACTTATTACATGTTTGCACTGG
	R: TAATAGCCATAATAATAGCCATGTT
hps1 Q140* OT	F: AGTGGCCGATGAGTGTAAGG
	R: ACGCACCTCCACTAAAAAGC
hps1 Q140* OF1	F: TTGTTGTTTACCTGCGGGTGCC
	R: AGCATTGTGTTCTACTGCTCCAC
hps1 Q140* OF2	F: AGCACGCTGTGCTCTCAGCA
	R: AACTTTCTCAAGCTCATCTGAGTG
hps1 Q140* OF3	F: TCCGGGTGAGGTGGCACAGTTGAA
	R: TCTTTAGGTTTTCACCCTCAGACCA
